# Assessment of N-terminal pro-B-type natriuretic peptide level in screening for atrial fibrillation in primary health care

**DOI:** 10.1371/journal.pone.0212974

**Published:** 2019-02-26

**Authors:** Faris Ghazal, Holger Theobald, Mårten Rosenqvist, Faris Al-Khalili

**Affiliations:** 1 Karolinska Institute, Department of Clinical Sciences, Cardiology Unit, Danderyd University Hospital, Stockholm, Sweden; 2 Karolinska Institute, Department of Neurobiology, Care Sciences and Society, Stockholm, Sweden; Scuola Superiore Sant'Anna, ITALY

## Abstract

**Background:**

Atrial fibrillation (AF), an important cause of thromboembolic events, is often silent and intermittent, thus presenting a diagnostic challenge. The aim of this study was to assess whether the plasma level of N-terminal pro-brain natriuretic peptide (NT-proBNP) is related to the presence of AF and thereby might be used to facilitate screening programs for AF in primary care.

**Methods:**

This was a cross sectional screening study. A population of 70–74-year-old individuals registered at a single primary care center in Stockholm were invited to AF screening. Intermittent ECG recording, 30 seconds twice a day using a hand-held device over 2 weeks, was offered to participants without previously known AF. Of the 324 participating persons, 34 patients had already known AF and 16 new cases of AF were detected by screening. Plasma NT-proBNP was measured in patients with previously known AF, newly detected AF, and 53 control participants without AF.

**Findings:**

The median NT-proBNP was 697 ng/L in patients with previously known AF, 335 ng/L in new cases of AF, and 146 ng/L in patients without AF. After adjustment for several clinical variables and morbidities, the differences of median NT-proBNP levels were statistically significant between cases of previously known AF and new cases of AF as well as between new cases of AF and those without AF. The area under receiver operating characteristic curve of detection of new AF was 0.68 (95% CI 0.56 to 0.79) yielding a cut-off point of 124 ng/L with 75% sensitivity, 45% specificity, and 86% negative predictive value.

**Conclusions:**

The NT-proBNP plasma levels among patients with known AF are higher than those with newly detected AF, and the latter have higher levels than those without AF. Therefore NT-proBNP might be a useful screening marker for the detection of AF and its persistence.

## Introduction

Atrial fibrillation (AF) is a common cardiac arrhythmia carrying a high risk for ischemic stroke [1]. Oral anticoagulant therapy reduces the risk of stroke by at least 60% and is recommended for most patients with AF [1]. Therefore, early identification of AF and initiation of oral anticoagulant therapy might prevent stroke.

Opportunistic screening for AF using pulse–palpation is recommended in persons above the age of 65 according to the European Society of Cardiology guidelines [1]. By single time-point screening of individuals aged 65 years and older, 1.4% new AF cases can be detected [2]. However, AF can be difficult to diagnose because it might be intermittent and asymptomatic [2].

Screening for AF among individuals aged 70–74 years in primary care using intermittent electrocardiogram (ECG) recordings for 2 weeks yielded 5.5% individuals with newly detected AF [3].

N-terminal pro B-type natriuretic peptide (NT-proBNP) can be used as a biomarker for predicting the development of AF [4,5,6], stroke [7,8,9,10] and mortality NT-proBNP in patients with AF.

The role of NT-proBNP in screening for AF in primary care has not been studied. The aim of this study was to evaluate the usefulness of NT-proBNP in systemic screening for AF in primary care.

## Methods

https://www.protocols.io/view/the-feasibility-and-outcome-of-atrial-fibrillation-m2fc8bn.

### Screening procedure

The study population was selected from a previous cross sectional screening study for AF [3], and the design of this screening study has been published previously^3^. Briefly, the target population of the study was 415 individuals 70–74 years old who were registered at a single primary care center (PCC). Patients with previously known AF were invited for routine physician visits in the PCC, and individuals without previously known AF and who visited the PCC for consultations for any reason during the one-year inclusion period were also invited to participate in the screening program. The remaining individuals who did not visit the PCC during the inclusion period received a written invitation to participate.

Participants received written and oral information about the study, and they gave their informed and written consent to participate. The responsible physician took the participants’ medical histories, including their current medications, and performed a general medical examination that included blood pressure measurement and fasting plasma glucose. Participants without previously known AF were examined with a 12-lead ECG. When the ECG did not show AF, intermittent handheld ECG (Zenicor) recordings were made for 30 seconds twice a day, and in case of palpitations recordings were made for at least two weeks. When handheld ECG findings showed AF or any other suspected pathological finding, the ECG was re-examined by an experienced cardiologist in order to confirm the diagnosis. Participants with unclear or uninterpretable ECG were further investigated with Holter ECG recordings. Oral anticoagulant treatment was offered to patients with newly detected AF.

**Fig 1:**
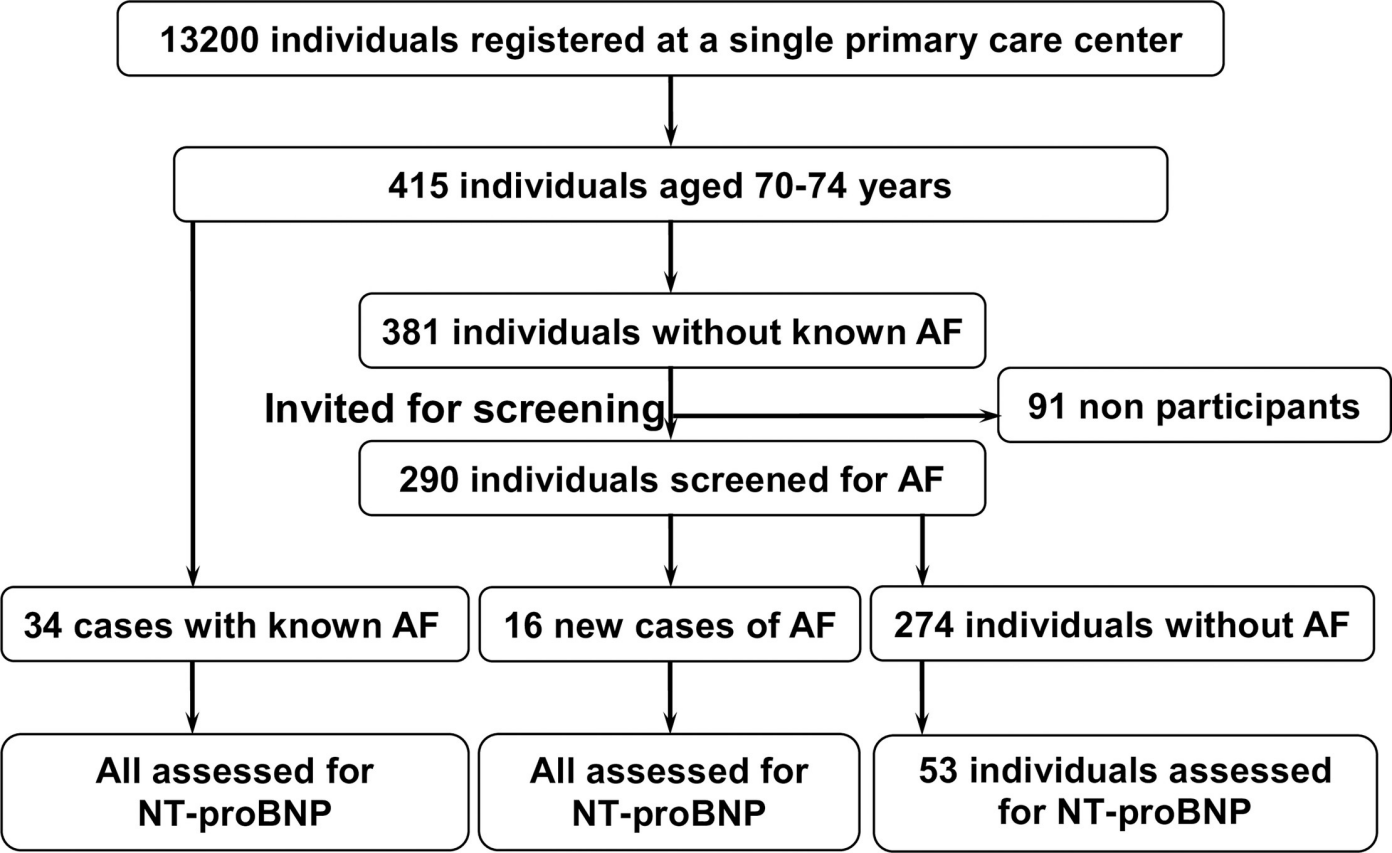
Flow chart illustrating the screening procedure.

Plasma NT-proBNP levels were evaluated at the end of the screening study. NT-proBNP was analyzed for all new cases of AF and all known cases of AF. Of those individuals with no detected AF, a non-randomly selected group of 53 individuals (18%) were also checked for NT-proBNP through venous blood sampling (Fig 1). Plasma NT-proBNP levels were measured using high-sensitivity sandwich (two monoclonal antibodies [11]) electrochemiluminescence immunoassays (Roche Diagnostics GmbH Elecsys automated clinical analyzer for NT-proBNP assay [12]). Plasma creatinine was analyzed at the same time as NT-proBNP, and the Lund-Malmö equation was used to estimate the glomerular filtration rate [13].

### Statistical analyses

Categorical data were summarized by counts and percentages. For all continuous variables, visual inspection of histograms and the Shapiro–Wilk’s test were used to assess the deviation from a normal distribution. Normally distributed continuous data are reported as means with standard deviations, whereas non-normally distributed data are reported as medians with interquartile ranges. The CHA2DS2-VASc score [14] was regarded as ordinal data. Fisher’s exact test was used to analyze categorical variables. For comparison of the means and the medians of continuous variables between two groups, Student’s t-test and the Mann–Whitney U-test were used, respectively. For comparisons of the means and the medians of continuous variables between three groups, analysis of variance and Kruskal–Wallis tests were used, respectively.

Multivariable analysis was performed using binary logistic regression to test the association between AF and risk factors. The results are presented as odds ratios with 95% confidence intervals. NT-proBNP data were not normally distributed and were therefore normalized using natural logarithmic transformation, and the logarithmic mean was used in the regression test.

Receiver operating characteristic analysis was performed, and the discriminative ability was assessed by the area under curve (AUC). Youden’s index was calculated to determine whether it could be used to find a clinically relevant cut-off for NT-proBNP. The specificity balanced with the desired high sensitivity for different cut-offs was then studied to determine a clinically relevant cut-off value.

For all tests, a p-value < 0.05 was considered statistically significant. All analyses were performed using the Stata statistics program version 10.

### Ethics approvals

This study was performed in accordance with the Helsinki Declaration and was approved by the Ethics Committee of Stockholm (DNR 2014/2061–31 and 2017/129-32). All patients gave their oral and written consent.

## Results

### Comparison between groups with consideration for NT-proBNP assessment

Plasma NT-proBNP was measured in 103 individuals of the 324 participants in the previous screening study. The analysis included 16 new cases of AF and all 34 known cases of AF. Altogether 53 (18%) individuals were selected from the 274 individuals with no detected AF and had their NT-proBNP levels measured. The clinical characteristics of those 53 individuals did not differ significantly from the remaining 221 individuals with no detected AF (Table 1).

**Table 1:** Comparison between individuals with no detected AF who were assessed for NT-proBNP and individuals with no detected AF who were not assessed for NT-proBNP.

	NT-proBNP measurement 53 individuals	No NT-proBNP measurement 221 individuals	P-value
Age, mean years (SD)	72.2 (1.5)	71.8 (1.5)	0.060
Women, N (%)	31 (58.5)	117 (52.9)	NS
Non-Swedish birth country, N (%)	10 (18.9)	48 (21.7)	NS
Civil state living alone, N (%)	23 (43.4)	121 (54.8)	0.091
Alcohol consumption, median glasses/week (IQR)	1 (0,4)	2 (0,6)	0.128
Smoking: Current smoker, N (%)	17 (32.1)	81 (36.7)	NS
Previous smoker, N (%)	25 (47.2)	103 (46.6)	
Never smoker, N (%)	11 (20.8)	37 (16.7)	
CHA2DS2-VASc score, median (IQR)	3 (2,3)	3 (2,3)	NS
CHA2DS2-VASc score, mean (SD)	2.9 (1)	2.8 (1)	NS
Congestive heart failure, N (%)	2 (3.8)	5 (2.3)	NS
Hypertension, N (%) post-screening	39 (73.6)	168 (76)	NS
Diabetes mellitus, N (%) post-screening	13 (24.5)	46 (20.8)	NS
Previous stroke and/or TIA, N (%)	5 (9.4)	16 (7.2)	NS
Vascular disease^§^, N (%)	6 (11.3)	24 (10.9)	NS
Chronic obstructive pulmonary disease, N (%)	3 (5.7)	23 (10.4)	NS
Sleep apnea, N (%)	1 (1.9)	4 (1.8)	NS
Dementia, N (%)	1 (1.9)	11 (5)	NS
History of malignancy, N (%)	11 (20.8)	43 (19.5)	NS
Mobility class: No problems with walking	39 (73.6)	179 (81)	NS
Some problems with walking	14 (26.4)	40 (18.1)	
Bedridden	0 (0)	2 (0.9)	
Self-care class: No problems with self-care	52 (98.1)	217 (98.2)	NS
Some problems with self-care	1 (1.9)	2 (0.9)	
Inability to wash or dress self	0 (0)	2 (0.9)	
Class of usual activities (housework or leisure): No problems	48 (90.6)	200 (90.5)	NS
Some problems	5 (9.4)	19 (8.6)	
Inability to perform usual activities	0 (0)	2 (0.9)	
NYHA functional class: 1	34 (64.1)	151 (68.3)	0.162
2	16 (30.2)	67 (30.3)	
3	3 (5.7)	3 (1.4)	
4	0 (0)	0 (0)	
Health assessment score, median (IQR)	80 (70,90)	90 (75,90)	0.183
Body weight, mean kg (SD) women	67.5 (13.9)	68.7 (13.4)	NS
Body weight, mean kg (SD) men	90.8 (21.4)	87.1 (14.3)	NS
Height, mean cm (SD) women	160.4 (7.2)	161.4 (6.8)	NS
Height, mean cm (SD) men	176.3 (7.3)	175.5 (7.2)	NS
BMI, median kg/m^2^ (IQR) women	26.6 (22.0, 29.6)	25.9 (23.1, 29.0)	NS
BMI, median kg/m^2^ (IQR) men	28.4 (25.2, 31.8)	28.1 (25.9, 30.4)	NS
BP, mean mmHg (SD) systolic	145.5 (21.5)	146.5 (22)	NS
BP, mean mmHg (SD) diastolic	80 (10.7)	83 (12.1)	0.104
Beta blocker, N (%)	17 (32.1)	65 (29.4)	NS
Loop diuretic, N (%)	3 (5.7)	13 (5.9)	NS
Angiotensin receptor antagonist, N (%)	13 (24.5)	51 (23.1)	NS
Angiotensin-converting enzyme inhibitor, N (%)	13 (24.5)	53 (24)	NS
Non-loop diuretic, N (%)	14 (26.4)	45 (20.4)	NS
Calcium antagonist, N (%)	10(18.9)	46(20.8)	NS
Statin, N (%)	21(39.6)	66(29.9)	0.115
Antidiabetic drug, N (%)	12(22.6)	34(15.4)	0.144
Acetylsalicylic acid, N (%)	13(24.5)	42(19)	NS
Anti-depressive agent, N (%)	6(11.3)	20(9.1)	NS

SD, standard deviation; IQR, interquartile range; TIA, transient ischemic attack; NYHA, New York Heart Association; BP, blood pressure; NS, non-significant p-values above 0.2.

Student’s t-test was used to compare two means, the Mann–Whitney U-test was used to compare two medians, and Fisher’s exact test was used to compare two categories.

### NT-proBNP and AF (S1 Table)

**Fig 2:**
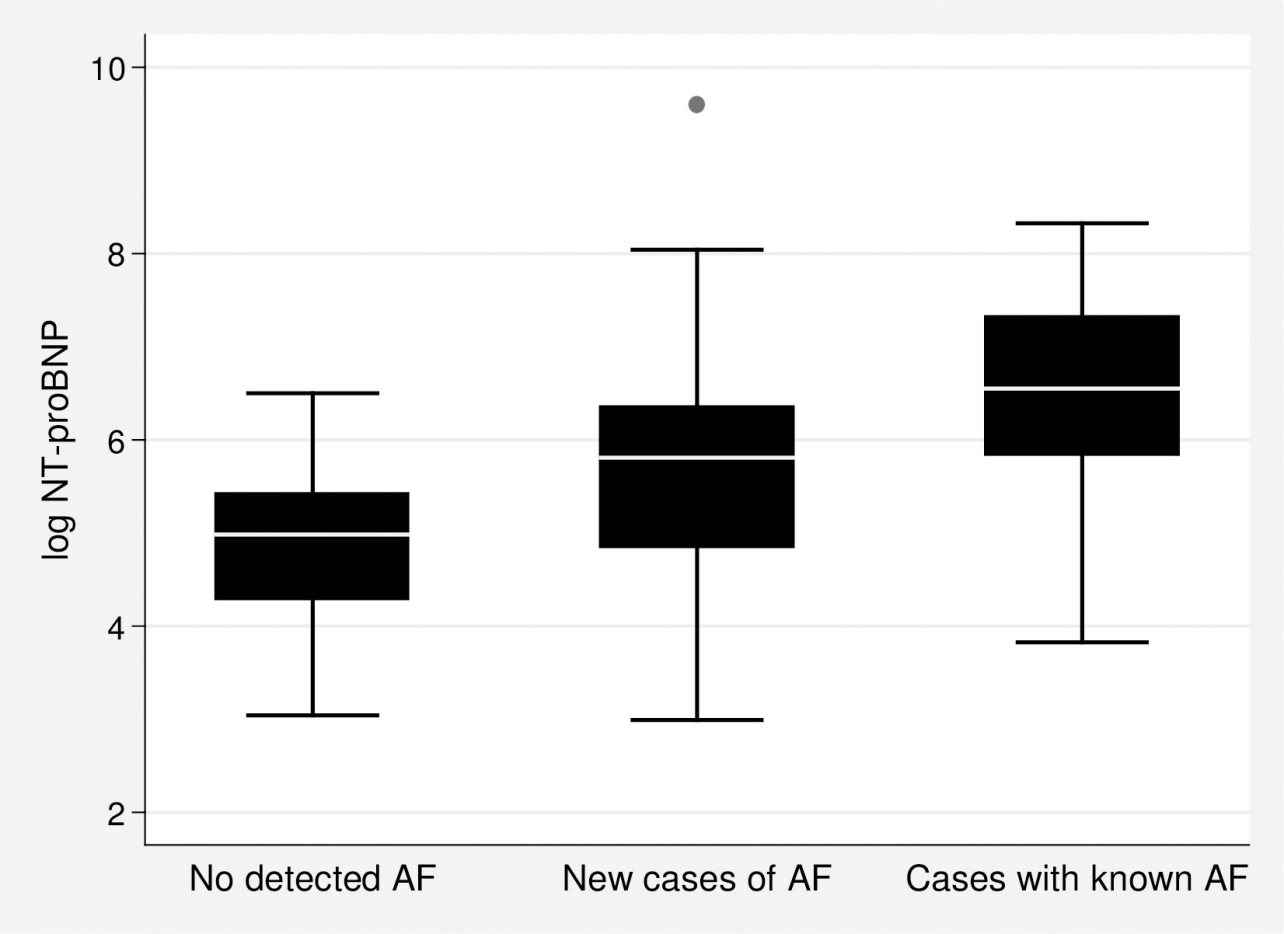
Box-plot showing natural logarithm-transformed NT-proBNP values for different AF groups.

New cases of AF had significantly higher NT-proBNP levels (median 335 ng/L (IQR (129, 575)) compared with individuals with no detected AF (146 ng/L (IQR 77, 239), p = 0.033). Moreover, cases with known AF had a significantly higher NT-proBNP level (median 697 ng/L (IQR 344, 1508)) compared with individuals with newly detected AF (p = 0.026) and compared with individuals with no detected AF (p < 0.001) (Table 2, Fig 2).

**Table 2:** Variables with statistically significant differences regarding AF among participants who were assessed for NT-proBNP.

	Without AF, 53 persons	P-Value new AF vs no AF	New AF,16 patients	P-Value known AF vs new AF	Known AF, 34 patients	P-Value known AF vs no AF
NT-proBNP ng/L Median (IQR)	146 (77, 239)	**0.033**	335 (129, 575)	**0.026**	697 (344, 1508)	**<0.001**
NT-proBNP ng/L Mean (SD) †	191 (155)		1381 (3652)		1164 (1153)	
eGFR, median mL/min/1.73 m^2^ (IQR)	72.7(65.8,80.6)	**0.014**	65 (58.9,71.4)	NS	65.6 (58.6,83.8)	**0.044**
CHA2DS2-VASc score Median (IQR)	3 (2,3)	NS	3 (2,3)	**0.048**	4 (3,5)	**0.007**
CHA2DS2-VASc score Mean (SD) †	2.9 (1)		2.9 (1)		3.8 (1.8)	
Congestive heart failure, N (%)	2 (3.8)	NS	1 (6.3)	**0.019**	14 (41.2)	**<0.001**
Previous stroke and/or TIA, N (%)	5 (9.4)	NS	0 (0)	**0.043**	9 (26.5)	0.07
Chronic obstructive pulmonary disease, N (%)	3 (5.7)	NS	2 (12.5)	NS	9 (26.5)	**0.01**
Sleep apnea, N (%)	1 (1.9)	**0.036**	3 (18.8)	NS	2 (5.9)	NS
Systolic BP, mean mmHg (SD)	145.5 (21.5)	NS	146.4 (15.8)	**0.027**	134.7‡ (17.4)	**0.016**
Diastolic BP, mean mmHg (SD)	80 (10.7)	**0.007**	88.9 (13.1)	NS	85.9 (10.1)	**0.012**
Body weight, mean kg (SD)	77.2 (20.7)	0.176	85.1 (19.3)	NS	86.6 (14.2)	**0.022**
Body weight, mean kg (SD) women	67.5 (13.9)	NS	72.4 (19.1)	NS	79.1 (9.5)	**0.007**
BMI, median kg/m^2^ (IQR) women	26.6 (22,29.6)	NS	23.5 (22.6, 31.7)	NS	30.2 (27.9, 32.9)	**0.013**
Beta blocker, N (%)	17 (32.1)	NS	6 (37.5)	**<0.001**	30 (88.2)	**<0.001**

SD, standard deviation; IQR, interquartile range; TIA, transient ischemic attack; BP, blood pressure; eGFR, estimated glomerular filtration rate; non-significant p-values above 0.2.Student’s t-test was used for comparison of means, the Mann–Whitney U-test was used for comparison of medians, and Fisher’s exact test was used for comparison of categories.

† Median comparison more appropriate and used instead of mean comparison

‡ lower systolic BP probably as a result of more beta-blocker treatment for AF.

### NT-proBNP and type of AF

The majority (68%) of cases with known AF had non-paroxysmal AF, compared to 37% of new cases. Of the 50 cases with AF in both known and new cases of AF, there were 29 cases of non-paroxysmal AF and 21 cases of paroxysmal AF. Cases with non-paroxysmal AF had a significantly higher NT-proBNP level (median 866 ng/L (IQR 579, 2478)) compared to cases with paroxysmal AF (208 ng/L (IQR 141, 396)) (p < 0.001).

### NT-proBNP as a predictor for AF

In addition to the differences in the NT-proBNP levels among AF groups, the new cases of AF had greater prevalence of sleep apnea, higher diastolic blood pressure, and lower estimated glomerular filtration rate (eGFR) than cases with no detected AF. Cases with known AF had a greater prevalence of heart failure and history of stroke and higher CHA2DS2-VASc score than new cases of AF. Compared with cases with no detected AF, cases with known AF had a greater prevalence of heart failure and chronic obstructive pulmonary disease, higher CHA2DS2-VASc score, higher diastolic blood pressure, and lower eGFR (Table 2). Women with known AF had higher body mass index compared with women with no detected AF.

In the final multiple logistic regression model for the likelihood of detecting new AF, the NT-proBNP logarithmic mean and diastolic blood pressure were significantly associated with AF detection with an odds ratio and 95% CI of 2.29 (1.11 to 4.69) for log NT-proBNP and 1.08 (1.02 to 1.15) for diastolic blood pressure. In the final multiple logistic regression model for the association between known AF and new AF (the reference group), only heart failure was significantly associated with known AF with an odds ratio and 95% CI of 10.5 (1.24 to 89.92).

**Fig 3:**
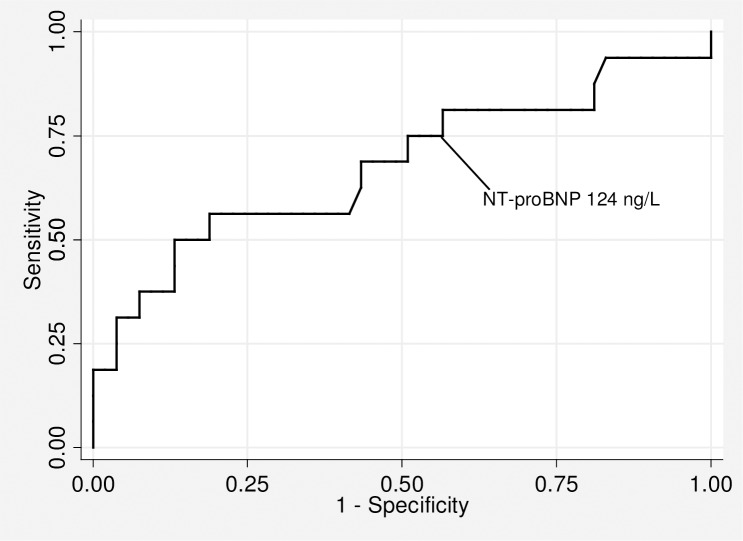
Receiver operating characteristic curve of NT-proBNP plasma level for the detection of new cases of AF.

Consequently, in the final multiple logistic regression model for the association between known AF and those with no detected AF (the reference group), heart failure, NT-proBNP logarithmic mean, and diastolic blood pressure were significantly associated with known AF with an odds ratio and 95% CI of 48 (3.7 to 622.7) for heart failure, 6.68 (2.66 to 16.77) for log NT-proBNP (Fig 3), and 1.12 (1.04 to 1.21) for diastolic blood pressure.

The AUC for discrimination of newly detected AF for NT-proBNP alone was 0.68 (95% CI 0.56 to 0.79) and for diastolic blood pressure alone was 0.71 (95% CI 0.60 to 0.83), but this difference was not statistically significant (p = 0.8). Adding diastolic blood pressure to NT-proBNP increased the AUC to 0.72 (95% CI 0.57 to 0.87). However, the differences in the AUC of the combined model versus either diastolic blood pressure alone or NT-proBNP alone were not statistically significant (p = 0.23 and 0.7, respectively).

To determine a cut-off level for NT-proBNP for AF screening, we considered at least 75% sensitivity as optimal in this non-acute setting. This resulted in a cut-off of 124 ng/L with a specificity of 45% and a negative predictive value of (24/28) 86%. Using this cut-off value, 28/69 (41%) of the patients would not have had to undergo screening by intermittent ECG. When Youden’s index was used instead to find the optimal cut-off for NT-proBNP, this resulted in a NT-proBNP cut-off value of 570 ng/L with a sensitivity of 31% and a specificity of 96%.

Similar results using a cut-off diastolic blood pressure of 81 mmHg resulted in a sensitivity of 75% and a specificity of 53% with a negative predictive value of (33/37) 89%. Again, using a cut-off based on diastolic blood pressure, fewer individuals (32/69) 46% would need to go through AF screening with intermittent ECG recordings.

## Discussion

This was the first screening study for AF in primary care where the role of NT-proBNP was evaluated. Participants in the screening program for AF who had newly detected AF had significantly higher NT-proBNP levels than individuals without AF. An NT-proBNP cut-off of 124 ng/L provided a reasonable balance between specificity and high sensitivity.

### Comparison between groups with consideration for NT-proBNP assessment

There was low risk for selection bias among those who were selected from the screened population because all cases of new AF and known AF were included in the NT-proBNP assessment. Among the group of no detected AF, there were no statistically significant differences between those who were assessed for NT-proBNP and those who were not.

### NT-proBNP and AF

A previous cohort study [5] showed that cases with incipient AF with a mean age 72 years had a higher NT-proBNP level (271 ng/L) than those without AF (93 ng/L). Similarly, a pilot screening study [15] using 7-day Holter monitoring showed higher NT-proBNP levels in cases with newly detected AF compared to those with no AF. In another screening study [6] using intermittent ECG among 75–76 year olds, NT-proBNP levels were 472 ng/L, 330 ng/L, and 171 ng/L among known AF, newly detected AF, and no AF cases, respectively. The results of our study confirm these previous results.

Moreover, NT-proBNP is an independent predictor for the development of cardioembolic stroke [7,9], and therefore NT-proBNP can be used for the detection of AF and as a risk assessment for the development of stroke [10]. Detection of many cases of AF with high NT-proBNP and high stroke risk might override the risk of missing a few cases of AF with low NT-proBNP and relatively low stroke risk.

### NT-proBNP and type of AF

In our study, the NT-proBNP level was higher (866 ng/L) in cases of non-paroxysmal AF compared to cases of paroxysmal AF (208 ng/L). These findings are in accordance with findings from a previous screening study [6] with corresponding NT-proBNP levels of 847 ng/L and 227 ng/L, respectively. In another study [8], the strongest independent relationship with NT-proBNP was with type of AF followed by reduced creatinine clearance, heart failure, and age.

### NT-proBNP as a predictor for AF

There were no differences between detected cases of AF and those with no AF regarding the CHA2DS2-VASc score and CHARGE-AF [16] score without including the age factor because we studied the same age group of 70–74 years old. Our sample size might be too small to detect such differences. However, the previous scores were evaluated for incident AF as detected by usual care, while in our study we detected AF cases by a screening method. The role of NT-proBNP was evaluated in another screening study [6] for AF where only NT-proBNP level and obesity were predictors for AF after adjusting for clinical factors. In our study, NT-proBNP level and diastolic blood pressure were predictors for AF.

The AUC for detecting AF was 0.68 for NT-proBNP in our study compared with 0.64 in the previous screening study [6]. A 75% sensitivity for the detection of AF resulted in a cut-off of 124 ng/L in our study, which was almost identical to the cut-off of 125 ng/L in the previous study [6], and the specificity was similar at 45%. Thus, the results of using NT-proBNP in the detection of AF in our studied age group confirm the results of the previous screening study in other age groups. However, there is a need to study this cut-off point using larger multicenter trials with a wider age group.

In our study, patients with known AF already detected by usual health care had more prevalent heart failure than new cases of AF detected by screening in the same age group. This difference might be attributed to the duration of AF. However, known AF cases had more non-paroxysmal AF than new AF cases. Progress of paroxysmal AF into persistent/permanent AF with increasing NT-proBNP is associated with structural cardiac remodeling and the progression to heart failure with associated comorbidities, including stroke. Therefore, early detection of AF by screening and early preventive measures might prevent or delay the development of heart failure and associated comorbidities.

### Limitation

There are a few limitations to our study. The sample size was relatively small with low power to detect the associations between AF and many comorbidities. However, it could still detect the association between AF and NT-proBNP as well as the associations between AF and many comorbidities.

Although the AF detection was adjusted for many possible confounders, there is still a need to adjust the AF detection for other unmeasured confounders such as echocardiogram variables.

Using intermittent ECG recordings might underestimate the detection of AF. Intermittent ECG recordings detect a comparable proportion of AF as detection by five-day Holter monitoring. Prolonged Holter monitoring might detect a higher proportion of AF, but using intermittent ECG is easier and might be more acceptable by participants in the screening for AF than prolonged Holter monitoring.

Finally, the NT-proBNP cut-off of 124 ng/L derived in this study has not been validated in the study; however, the previous study [6] showed a similar cut-off of 125 ng/L.

### Conclusion

The plasma level of NT-proBNP was significantly higher among primary care patients 70–74 years old with newly detected AF compared to those without AF, and therefore NT-proBNP might be used as a useful marker for AF detection. Higher levels of NT-proBNP were found in patients with non-paroxysmal compared with paroxysmal AF and in patients with known AF compared with new cases of AF; therefore, NT-proBNP might also be a marker for AF burden and associated comorbidities.

## Supporting information

S1 Table Variables of the case reporting form. (XLSX)
